# Antiseptic Qualities of Saliva

**Published:** 1909-01-15

**Authors:** Henry C. Ferris


### ANTISEPTIC QUALITIES OF SALIVA.

Abstract from Report to New York State Dental Society, by Henry C. Ferris, and printed in December *Cosmos.*

In an endeavor to throw some light upon this particular phase of the subject, I addressed to sixty of the leading pathologists and bacteriologists of the colleges and hospitals throughout the world this letter:

*Dear Doctor,*—In behalf of science and the New York State Dental Society, I earnestly solicit your opinion upon three questions of vital importance, yet unsettled:

(1) Has the normal salivary secretion of man any inherent antiseptic quality?

(2) If it has, to what component part or parts do you attribute this action?

(3) What forms of bacteria are affected?

May I ask the favor of a reply to be incorporated in my report at our annual meeting in May 1908? Any courtesy you may extend will be appreciated by the six thousand dentists in the Empire State.

HENRY C. FERRIS, Correspondent

DR. G. V. BLACK: An answer to the first is all that is necessary from me, and that is an emphatic "No." I suppose this question is prompted by the work that has been going on with reference to the immunity and susceptibility of persons from decay of the teeth. Some persons seem to have the thought that the micro-organisms of caries are not growing in the mouths of immune persons.

As I have cultivated micro-organisms a great deal, and especially in relation to this matter, I wish to say distinctly that the same micro-organisms are found growing in the mouths of persons immune to decay as are found in the mouths of persons who are susceptible to decay and whose teeth are decaying rapidly, and the same micro-organisms will grow in the saliva of either one when used as a cultivating medium. The questions of susceptibility and immunity are not related to the question of antiseptics, but the question is in some degree of a similar nature to the development of antitoxins in diphtheria, for instance, or in other microbic diseases. It now seems probable that the influence which prevents caries in immune persons is different in some respects from the antitoxins. But just as antitoxins prevent disease by neutralizing the poisons produced by the micro-organisms of that particular disease, so in caries, the effect of the acid produced by micro-organisms is prevented from acting to produce caries.

FANEUIL D. WEISSE, M.D., professor of anatomy, New York College of Dentistry:

All lesions of the buccal parietes with a buccal aspect are rarely, if ever, complicated by the occurrence of septicemia therefrom. All such lesions are always attended by an increased secretion from the salivary glands, and from the mucous glands of the cavity.

The lesion of wound or exposed lacerated tissues, as an alveolus after the extraction of a tooth, would require at the skin surface careful antiseptic treatment to prevent infection and possible septicemia; to be sure, pyogenic bacteria are effective in producing pus in the alveolus, but septicemia never occurs.

A fracture of the body of the mandible is always compound with a buccal aperture of the lesion leading to the line of fracture; while supperation occurs in these cases, the pus flows from the buccal aperture, but while no precautions are taken to prevent it, general infection never occurs.

A compound fracture with a skin aperture of the lesion leading to the line of fracture requires the utmost antiseptic precautions to prevent infection and septicemia.

What is it that affords this immunity from the occurrences of septicemia from lesions with a buccal aspect, which if they had a skin aspect would require the most careful antisepsis to prevent septicemia?

Need we look farther than to ascribe the protection, when we note that these secretions are increased in all cases of lesions of the parietes with a buccal aspect?

The oral surgeon must naturally leave the positive explanation of the above clinical facts, which are presented to him, to the bacteriologist and chemist to determine.

LEO. F. RETTGER, bacteriologist, Yale University.

In reply to your letter of inquiry regarding the possible antiseptic action of normal salivary secretions, I would say that I have no decided opinions as yet, though the matter has been of considerable interest to me for some time.

(1) According to Clairmont (*Wien. klin. Woch.*, 47, 1906), saliva has an antiseptic action on the mouth bacteria. He claims that the reaction from the parotid glands is distinctly evidenced along this line. I am inclined to believe such an inherent action of the saliva to be at least a probability, though I have no data to offer. It seems that with all the chances of infection of the mouth and nose (injury to the gum, palate, lips, etc., and the persistent nose picking that so many people indulge in) that injury would be much more common than it really is if it were not for some sort of protective action on the part of the saliva or mucous lining.

(2) Some six or seven years ago Ashing claimed that the mucin has a decided antiseptic action. I tried some experiments along these lines, but could reach no definite conclusions.

(3) I have nothing definite to offer here, but would not be surprised if such action were more or less marked toward ordinary pus organisms as well as the tetanus and other blood poisoning-bacteria.

F. BARNARD LEROY, New York city:

First Question. Yes, preventive in nature, but not destructive as in the sense of a germicide.

Second Question. Lime, magnesium, and ptyalin.

Third Question. Vegetative forms are prevented from undergoing rapid development, these spore-bearing bacteria being prevented from joining into the vegetative form so long as saliva is normal.

DR. J. P. MICHAELS, Paris, France:

I surmise that a difference of proportions in the components part is an important factor in dyscrasic saliva; that an excess of one constituent corresponds with the insufficiency of another; that the addition of a chemical element, a soluble acid, or alkaline product, will modify the chemical relation of the normal saliva, also that bacterial viscosine or organic toxins, likewise the insufficiency of the mineral elements—which are the inciting factors of the vital activity of enzymes, and of organic life—must, and does, modify the essential albuminoid elements.

We know that gingivo-dental affections are related in general to vitiated muco-slime, intolerant to the tissues.

We know of periods and conditions of saliva where the dental organs are immune to active dental decay; likewise we note dental lesions or abrasions of tissue in the case of the same saliva if charged with a greater quantity of the sulfocyanid (aldehyd) than normal.

We also understand that a high percentage of ptyalin digests the decalcified or decomposed ossein of carious teeth. The conditions here related induce a conviction of definite chemical action—in a living liquid secretion—the study of which by analytic determination of its dominant factors must give important and conclusive results.

Edward C. Kirk:

I have for a number of years kept a close watch upon the work done in connection with the composition of the saliva, and I have been especially interested in the studies which have been made by various observers to discover whether the normal salivary secretions of man contain any inherent antiseptic properties. I have made no direct experiments in this line myself, my work having been directed to other phases of saliva study, and my opinion with reference to the existence or absence of antiseptic properties in the saliva is based wholly upon the studies of others more competent to pursue that class of work. The most notable investigations of this question have been made by Dr. Arthur C. Hugenschmidt, of Paris, and by the late Professor W. D. Miller. The results of these researches, as I interpret them, are that the saliva contains no antiseptic properties. I have never from the beginning seen any reason to think that the saliva should contain antiseptic properties. The whole question as to the properties of the saliva, which result in immunity to dental diseases on the one hand, and insusceptibility on the other, is in my opinion not a question of the presence or absence of a definite antiseptic agency in the saliva, but is a question rather of fundamental conditions in chemical composition of the saliva, which in one instance make it a suitable pabulum for the growth of certain types of micro-organism, and in the other instance the absence of certain elements of composition render it an unfit culture medium for certain forms of micro-organism.

I have been able in the study of the saliva of carious susceptibles to discover by chemical test the presence of a dissolved carbohydrate which I believe is a component of the saliva as it issues from the gland, and not a carbohydrate which is derived from food debris. In the saliva of carious immunes I did not get the same reaction. My belief therefore is that the composition of the saliva with respect to its suitability as a pabulum for the growth of bacterial forms is the key to the solution of the question of susceptibility and immunity, and that it is not a question of the presence of an antiseptic agent.

We very well know that if two vessels, one containing, for example, beef juice, and the other containing fruit juice, as for instance sweet cider, are exposed to the atmosphere under exactly the same conditions whereby a mixed infection occurs in both, in the course of time the first will undergo decomposition by a putrefactive process and become almost a pure culture of a saprophytic organism which decomposes beef extract with the formation of acid end-products; those bacteria, in each case, which are unable to find their proper nutriment in the two culture media respectively, perish, the principle being that the culture media in each instance possess a selective quality for the type of germs best able to thrive under the particular conditions induced by the composition of the two fluids. My belief is that the same principle holds good with respect to mouth infections, and that the type of infection is governed almost exclusively by the composition of the oral fluids and the tissue juices. Acting upon this theory I have been giving treatment in certain cases with a view to changing the salivary compositions, my work in this particular having been addressed particularly to erosion cases, and I am glad to be able to report that I have had a marked success in changing the composition and incidentally the reaction of the mixed oral secretions in erosion cases, so that instead of an acid exudate I have been able to bring about a neutrality or alkaline condition of the buccal mucus in from ten days to two weeks' time. I believe that the same alteration can be made in the treatment of the carious susceptibles upon the same general plan.

DR. FERRIS: In summing up these replies, your Correspondent concludes that while a number of the most eminent scientists who have been working in this field agree that the normal salivary secretions may not contain an ingredient that can be eliminated, to be considered a germicide, their scientific deductions are to the effect that in and through its chemical and physiological fermentive action, this secretion, in normal balance of its properties, inhibits the growth of bacteria; therefore, if this normal balance of chemical and physiological ferments exists, the enzymes and anti-enzymes will protect the oral cavity from the pathogenic bacteria.

If we accept this conclusion as scientific it must necessarily stimulate our effort to further study the physiological changes that occur, that we may be able to diagnose pathological conditions from our findings.

